# Twin Transformation in Cardiothoracic Surgery: The Convergence of Digital Innovation and Sustainability

**DOI:** 10.3390/jcdd13030122

**Published:** 2026-03-07

**Authors:** Vasileios Leivaditis, Roman Gottardi, Andreas Antonios Maniatopoulos, Francesk Mulita, Charalampia Pylarinou, Spyros Papadoulas, Konstantinos Nikolakopoulos, Ioannis Panagiotopoulos, Efstratios Koletsis, Manfred Dahm, Anastasios Sepetis

**Affiliations:** 1 Department of Cardiothoracic and Vascular Surgery, Westpfalz Klinikum, 67655 Kaiserslautern, Germany; vnleivaditis@gmail.com (V.L.); roman.gottardi@gmail.com (R.G.); mdahm@westpfalz-klinikum.de (M.D.); 2 Department of Electrical and Computer Engineering, Democritus University of Thrace, 67100 Xanthi, Greece; amaniatopoulos@gmail.com; 3 Department of General Surgery, General Hospital of Eastern Achaia—Unit of Aigio, 25100 Aigio, Greece; 4 Department of Mechanical and Aeronautical Engineering, University of Patras, 26500 Patras, Greece; pylarinou@systserv.com; 5 Department of Vascular Surgery, General University Hospital of Patras, 26504 Patras, Greece; spyros.papadoulas@gmail.com (S.P.); konstantinosn@yahoo.com (K.N.); 6 Department of Cardiac Surgery, Hippokration General Hospital of Athens, 11527 Athens, Greece; mdgiapan@yahoo.gr; 7 Department of Cardiothoracic Surgery, General University Hospital of Patras, 26504 Patras, Greece; ekoletsis@hotmail.com; 8 Postgraduate Health and Social Care Management Program, Department of Business Administration, University of West Attica, 12244 Athens, Greece; tsepet@uniwa.gr

**Keywords:** cardiothoracic surgery, twin transformation, digital transformation, digital health, artificial intelligence, sustainability, digital twins, telemedicine, robotic surgery, green healthcare

## Abstract

Background: Cardiothoracic surgery is among the most technologically advanced and resource-intensive medical specialties, placing it at the intersection of rapid digital innovation and growing demands for environmental sustainability. Addressing these parallel pressures requires integrated strategies that reconcile clinical excellence with ecological responsibility. Methods: This narrative review synthesizes PubMed-indexed literature published over the past two decades, supplemented by relevant policy documents and guidelines. The review examines digital transformation and sustainability initiatives in cardiothoracic surgery through the lens of the twin transformation framework, which conceptualizes digitalization and sustainability as interdependent and mutually reinforcing processes. Results: Key domains of digital transformation include artificial intelligence and big data-driven decision-making, robotic and minimally invasive surgical techniques, digital twins and simulation-based training, telemedicine and remote monitoring, and interoperable electronic health records. Sustainability-related themes encompass the substantial environmental burden of operating rooms, green surgical practices, sustainable procurement, and hospital-level decarbonization strategies. Emerging evidence suggests that aligning digital technologies with sustainability objectives can improve clinical outcomes, enhance operational efficiency, and reduce environmental impact. However, current evidence is largely derived from pilot studies and single-center experiences. Conclusions: Twin transformation offers a coherent and forward-looking framework for the future evolution of cardiothoracic surgery, demonstrating that digital innovation and sustainability can be synergistic rather than competing goals. While significant challenges remain—including high implementation costs, limited long-term data, and fragmented regulatory frameworks—integrating digital health technologies with sustainable practices represents a promising pathway toward high-quality, efficient, and environmentally responsible cardiothoracic care.

## 1. Introduction

### 1.1. Cardiothoracic Surgery as a High-Complexity, High-Impact Medical Specialty

Cardiothoracic surgery occupies a distinctive position in contemporary medicine due to its exceptional clinical complexity and substantial resource requirements. Procedures such as heart transplantation, lung resection, valve repair or replacement, and coronary artery bypass grafting demand advanced technology, interdisciplinary expertise, and meticulous perioperative management. Long operative times, frequent reliance on extracorporeal circulation or cardiopulmonary bypass, and the use of highly specialized equipment characterize the specialty. As a result, cardiothoracic surgery is widely recognized as one of the most resource-intensive areas of healthcare delivery. At the same time, this technical sophistication has contributed to remarkable improvements in patient survival and quality of life, underscoring the need for a correspondingly advanced clinical infrastructure [1,2].

The operational environment of cardiothoracic surgery further reflects this dual nature of innovation and intensity. Operating rooms are equipped with state-of-the-art imaging systems, robotic platforms, and life-support technologies, while intensive care units provide prolonged postoperative monitoring and highly specialized care. However, these advances come at a considerable cost. The extensive use of sterile supplies, single-use instruments, and energy-intensive systems results in high operational expenditures and a substantial environmental footprint. Compared with many other surgical specialties, cardiothoracic operating rooms consume significantly more energy and generate large volumes of medical waste, extending the impact of each procedure beyond immediate clinical outcomes [1,2].

Beyond its technical demands, cardiothoracic surgery is also organizationally complex, requiring close coordination among surgeons, anesthesiologists, perfusionists, nursing staff, and rehabilitation specialists within integrated hospital pathways. Successful outcomes depend not only on surgical expertise but also on efficient teamwork, data exchange, and institutional support structures [3]. This convergence of clinical sophistication, technological dependence, high resource consumption, and systemic complexity positions cardiothoracic surgery as both a driver of medical innovation and a focal point in discussions surrounding sustainability and digital transformation in healthcare [4].

### 1.2. Rising Global Healthcare Challenges

The digitization of medicine is rapidly transforming healthcare systems worldwide, reshaping how care is delivered, monitored, and evaluated. The expanding availability of big data, advances in artificial intelligence, and the widespread adoption of telemedicine promise more accurate diagnoses, personalized treatment strategies, and improved operational efficiency across healthcare institutions. At the same time, the implementation of digital technologies introduces significant challenges, including integration with existing infrastructure, workforce training requirements, data protection concerns, and the risk of widening disparities between high- and low-resource healthcare settings [5,6,7]. For technology-dependent specialties such as cardiothoracic surgery, digitalization is therefore not a distant or optional development, but an immediate and unavoidable reality.

Concurrently, healthcare systems face mounting pressure to address their environmental impact amid the accelerating effects of climate change. Hospitals are among the largest energy consumers in the public sector, with operating rooms contributing disproportionately to greenhouse gas emissions through high energy demand, the use of anesthetic gases, and the generation of large volumes of medical waste. Due to prolonged operating times and reliance on advanced, energy-intensive technologies, cardiothoracic surgery exemplifies the tension between achieving optimal clinical outcomes and minimizing environmental harm. Increasingly, the international healthcare community recognizes that sustainability is not an optional adjunct to clinical excellence but a fundamental responsibility, given that healthcare systems are both affected by and contributors to climate change [8].

It is within this context that the concept of “twin transformation” has emerged, originating from industrial and policy frameworks and referring to the parallel pursuit of digital transformation and sustainability as mutually reinforcing strategies. Rather than treating technological innovation and environmental responsibility as separate objectives, the twin transformation approach emphasizes their integration, proposing that digital technologies can actively support ecological goals while enhancing clinical performance [5,6]. In healthcare, this perspective provides a structured response to the dual pressures of rapid digitalization and the urgent need for environmental stewardship, offering a coherent framework through which resource-intensive fields such as cardiothoracic surgery can align innovation with sustainability.

### 1.3. Rationale for Applying This Framework and Objective of the Review

Cardiothoracic surgery lies at the intersection of high technological dependency and substantial environmental impact, making it one of the medical specialties best suited for the application of the twin transformation framework. Historically, the field has been at the forefront of surgical innovation, from the development of cardiopulmonary bypass to the adoption of robotically assisted procedures and simulation-based training. Today, cardiothoracic practice increasingly relies on data-driven decision-making, precision imaging, and advanced perioperative monitoring, creating an ideal environment for the implementation of digital health technologies. At the same time, many cardiothoracic procedures rank among the most resource-intensive in medicine, frequently involving prolonged operating times, energy-intensive infrastructures, and extensive use of single-use equipment and consumables.

This combination of digital intensity and ecological burden underlines both the necessity and the opportunity for cardiothoracic surgery to lead the implementation of twin transformation principles. By deliberately aligning technological innovation with sustainability objectives, the specialty has the potential not only to reduce its environmental footprint but also to enhance patient outcomes and operational efficiency. In doing so, cardiothoracic surgery may serve as a model for other surgical disciplines seeking to reconcile clinical excellence with environmental responsibility [9].

Against this backdrop, the present review aims to examine how twin transformation is reshaping cardiothoracic surgery through the integration of digital health innovation and sustainability initiatives. Specifically, the objectives of this review include the following: (i) to summarize existing evidence on digital transformation in cardiothoracic surgery, including applications of robotics, artificial intelligence, digital twins, telemedicine, and electronic health records; (ii) to analyze sustainability challenges and practices relevant to the specialty, ranging from hospital-level decarbonization strategies to operating room waste management; and (iii) to identify areas in which digital and sustainable approaches intersect to generate synergistic benefits, an area in which preliminary but increasingly promising evidence is emerging [10,11].

**Figure 1:**
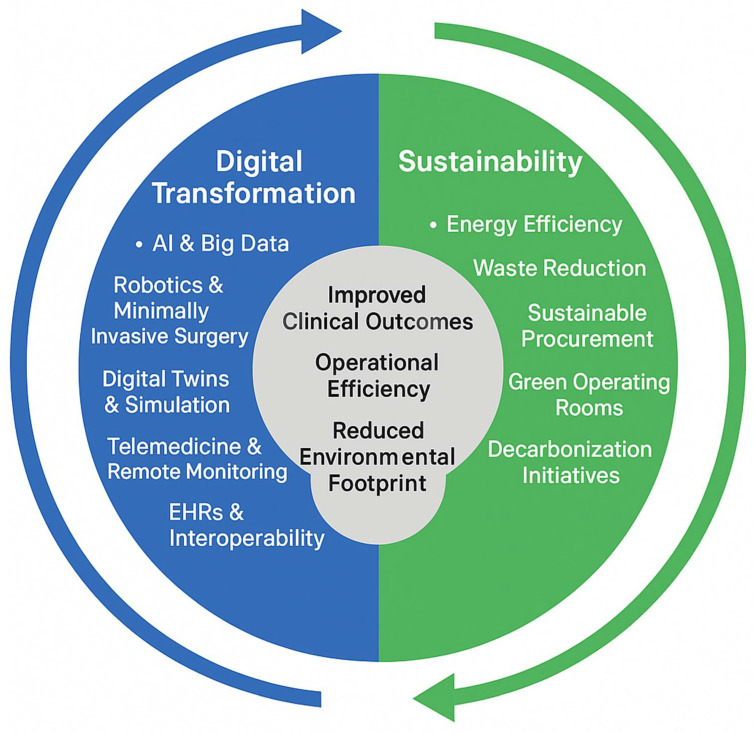
Conceptual framework of the twin transformation in cardiothoracic surgery.

To provide a comprehensive perspective, the scope of this review is intentionally broad and multidisciplinary, incorporating peer-reviewed clinical research, engineering studies, and relevant policy documents. By situating cardiothoracic surgery within the twin transformation paradigm, this work aims to inform future clinical practice, support policy development, and help define research priorities at the intersection of digital innovation and sustainability. The conceptual framework of the twin transformation in cardiothoracic surgery, illustrating the bidirectional and synergistic interaction between digital transformation and sustainability and their combined impact on clinical outcomes, operational efficiency, and environmental responsibility, is shown in Figure 1.

## 2. Materials and Methods

### 2.1. Study Design

This work was conducted as a narrative literature review aimed at synthesizing evidence from clinical, technological, and environmental domains relevant to cardiothoracic surgery. A narrative approach was deliberately chosen because the concept of twin transformation—the integrated pursuit of digital transformation and sustainability—is relatively recent and spans multiple disciplines, including medicine, biomedical engineering, health informatics, and health policy. In this context, a narrative format allows for conceptual integration, thematic synthesis, and critical interpretation of heterogeneous evidence, which would be difficult to capture through quantitative aggregation alone [12].

### 2.2. Literature Search Strategy

A structured literature search was performed using three major electronic databases: PubMed/MEDLINE, Scopus, and Web of Science, selected to ensure comprehensive coverage of biomedical research, interdisciplinary scientific literature, and policy-oriented publications. The search strategy combined controlled vocabulary terms and free-text keywords using Boolean operators.

Core search terms related to cardiothoracic surgery included “*cardiothoracic surgery*”, “*cardiac surgery*” and “*thoracic surgery*”. These were combined with terms reflecting digital transformation, such as “*artificial intelligence*”, “*machine learning*”, “*robotic surgery*”, “*digital twin*”, “*telemedicine*” and “*electronic health records*”, as well as sustainability-related terms, including “*sustainability*”, “*green surgery*”, “*environmental impact*” and “*climate change and healthcare*”.

In addition to database searches, the reference lists of included articles and relevant review papers were manually screened to identify additional studies not captured in the initial search.

### 2.3. Eligibility Criteria

Inclusion Criteria

- Publications were considered eligible for inclusion if they met the following criteria:
- Addressed cardiothoracic, cardiac, or thoracic surgery in relation to digital health technologies, sustainability, or both;
- Included original research articles, systematic or narrative reviews, clinical guidelines, consensus statements, or policy reports from recognized scientific or professional organizations;
- Employed quantitative, qualitative, or mixed-methods designs, reflecting the interdisciplinary nature of the topic;
- Were published primarily between 2005 and 2025, capturing contemporary developments in digital health and sustainability;
- Were written in English.

Earlier publications were selectively included when they provided essential historical context for landmark developments, such as the introduction of cardiopulmonary bypass, robotic surgery, or foundational environmental assessments of surgical practice [13].

Exclusion Criteria

Publications were excluded if they met the following criteria:

- Focused on surgical specialties unrelated to cardiothoracic surgery without clear relevance or transferability;
- Discussed digital health or sustainability in general healthcare settings without specific application to surgical or cardiothoracic practice;
- Consisted solely of opinion pieces, editorials, or commentaries lacking empirical evidence or analytical depth;
- Were non-peer-reviewed sources without institutional or professional endorsement;
- Addressed operating room sustainability or digitalization in a manner too generic to meaningfully inform cardiothoracic surgical practice.

### 2.4. Data Extraction and Synthesis

Data were extracted with emphasis on each study’s aims, methodological approach, key findings, and relevance to the twin transformation framework. Given the heterogeneity of study designs, outcomes, and disciplinary perspectives, findings were synthesized thematically rather than quantitatively.

The included literature was organized into four overarching thematic domains:

(a) Digital transformation in cardiothoracic surgery, encompassing artificial intelligence, robotics, telemedicine, digital twins, and data-driven decision support;
(b) Sustainability in cardiothoracic surgery, focusing on the environmental impact of operating rooms, green surgical practices, and hospital-level sustainability initiatives;
(c) Synergistic effects of twin transformation, highlighting areas where digital tools directly support sustainability objectives or where environmental imperatives stimulate technological innovation;
(d) Challenges, limitations, and future perspectives, addressing organizational, technical, economic, ethical, and regulatory barriers to implementation [14].

This thematic framework enabled the integration of diverse evidence into a coherent narrative, supporting a comprehensive analysis of how digital innovation and sustainability intersect within contemporary cardiothoracic surgery.

## 3. Digital Transformation in Cardiothoracic Surgery

### 3.1. Artificial Intelligence and Big Data

Artificial intelligence (AI) has rapidly emerged as a transformative force in cardiothoracic surgery, with applications spanning imaging, diagnostics, surgical planning, and perioperative management. AI-driven image analysis has demonstrated high accuracy in lung cancer staging, valve pathology assessment, and coronary artery disease evaluation, in many cases achieving performance comparable to or exceeding that of conventional radiological interpretation. In parallel, machine learning-based predictive models for perioperative risk stratification have gained prominence, with several studies reporting superior performance compared with traditional risk scores such as EuroSCORE II and the Society of Thoracic Surgeons (STS) models. Collectively, these findings illustrate a consistent trend toward improved clinical decision-making and more personalized risk assessment through data-driven approaches [15]. However, the apparent superiority of machine learning models over traditional risk scores must be interpreted cautiously, as many comparisons are conducted within retrospective datasets without uniform external validation. Whether these performance gains translate into consistent improvements in real-world clinical decision-making remains an open question.

Despite this rapid progress, the maturity of AI applications varies substantially across clinical domains. Imaging and diagnostic tools represent the most advanced and validated use cases, often supported by large, multicenter datasets that demonstrate reproducibility. In contrast, applications in surgical planning and intraoperative guidance remain largely confined to pilot studies or single-center experiences. While many of these investigations are limited by retrospective designs and selection bias, they nonetheless suggest that AI may contribute not only to clinical accuracy but also to resource efficiency, for example, by anticipating complications, optimizing patient flow, and supporting more targeted use of perioperative resources. However, widespread clinical integration remains constrained by persistent challenges related to data interoperability, standardization, and workflow integration. Even so, the overarching conclusion of the literature is clear: big data analytics and AI have the potential to enhance both patient outcomes and system-level efficiency by augmenting surgical expertise, refining patient selection, and potentially reducing unnecessary interventions [16].

Evidence from comprehensive systematic reviews further stresses the breadth of AI applications across the perioperative continuum. In a previous systematic review of AI in thoracic surgery, 36 relevant studies were identified, demonstrating that AI-driven imaging and radiomics tools improve pulmonary nodule detection, lung cancer staging, and lymph node metastasis prediction with accuracy exceeding that of conventional methods. Intraoperative innovations, including AI-assisted navigation and augmented reality systems, were associated with improved surgical planning and safety, while robotic-assisted thoracic surgery was linked to enhanced precision, reduced operative times, and faster postoperative recovery. Postoperatively, wearable devices and AI-based monitoring systems enabled more individualized follow-up and earlier detection of complications. Importantly, this review also highlighted unresolved issues, including algorithmic bias, insufficient multicenter validation, and ethical concerns related to data security and accountability, emphasizing the need for robust governance frameworks, explainable AI, and standardized validation pathways prior to widespread clinical adoption [17].

Similar conclusions were drawn in a systematic review focusing specifically on cardiac surgery, which synthesized evidence from 121 studies evaluating AI applications across the perioperative pathway. Machine learning models consistently outperformed conventional risk stratification tools in predicting mortality and postoperative complications, confirming the strong predictive capacity of AI-based approaches. Additional benefits were reported in surgical planning and intraoperative guidance through computer vision and augmented cognition systems, supporting the broader trend toward minimally invasive and robot-assisted procedures. In the postoperative setting, AI-enabled monitoring and complication prediction were associated with improvements in patient safety and potential reductions in healthcare costs. Nonetheless, persistent barriers—including data quality issues, algorithmic bias, limited external validation, and challenges in integrating AI tools into routine workflows—were identified as major obstacles, reinforcing the need for interdisciplinary collaboration, rigorous evaluation, and appropriate regulatory oversight [18].

Beyond cardiac surgery, AI is increasingly embedded in thoracic surgical practice, with applications ranging from non-small-cell lung cancer diagnosis and lymph node metastasis prediction to automated extraction of electronic medical record data and support for robotic-assisted thoracic surgery. These technologies show considerable promise for improving perioperative efficiency, surgical accuracy, and patient safety. However, concerns related to bias, data privacy, and ethical governance continue to limit broader implementation, highlighting the importance of transparent algorithms and secure data infrastructures, particularly when machine learning and natural language processing techniques are employed [19].

Recent studies further illustrate the clinical potential of AI-based imaging and predictive modeling. In the context of connective tissue disease-associated interstitial lung disease, AI analysis of high-resolution computed tomography demonstrated strong agreement with expert radiologists and superior performance compared with less experienced readers, enabling more reproducible assessment of subtle longitudinal changes in lung parenchymal patterns. These findings suggest that AI may enhance multidisciplinary decision-making by reducing inter-reader variability and improving disease monitoring in complex pulmonary conditions [20]. Similarly, deep learning frameworks such as Z-Net have achieved high diagnostic accuracy in thoracic disease classification using large chest X-ray datasets, reinforcing the role of AI as a valuable adjunct to computer-aided diagnosis systems in both clinical and high-throughput screening settings [21].

In the perioperative risk prediction domain, interpretable machine learning models have shown encouraging results. For example, an Extreme Gradient Boosting-based model combining echocardiographic, biochemical, and clinical parameters demonstrated good predictive performance for major adverse cardiovascular events in patients undergoing aortic valve replacement or coronary artery bypass grafting. By incorporating explainability techniques such as SHAP analysis, the model provided clinically intuitive insights into individual risk profiles, underscoring the importance of transparency and interpretability for clinical acceptance. Nevertheless, the limited sample size and retrospective design highlight the need for prospective, multicenter validation before such models can be reliably implemented in routine practice [22].

Despite encouraging findings, the current evidence base remains heterogeneous, with substantial variation in study design, patient populations, algorithm validation methods, and outcome definitions. Many studies are retrospective or single-center, and external validation across diverse healthcare systems is still limited, underscoring the need for prospective multicenter evaluation.

### 3.2. Robotics and Minimally Invasive Surgery

Robotic platforms and minimally invasive techniques have become increasingly integrated into cardiothoracic surgery, particularly in thoracic resections, mitral valve repair, and coronary artery bypass grafting (CABG). Compared with conventional open procedures, robotic approaches are consistently associated with shorter hospital stays, reduced postoperative pain, and faster recovery, leading to earlier return to baseline functional status. Beyond their clinical advantages, these outcomes carry important sustainability implications. Reduced length of stay translates into lower overall resource utilization, while enhanced surgical precision and smaller incisions are associated with fewer wound complications and infections, thereby decreasing the need for antibiotics, reinterventions, and hospital readmissions [23,24]. While these findings suggest potential advantages, much of the reported benefit may reflect institutional expertise, patient selection, and learning curve effects rather than inherent technological superiority. Moreover, the long-term cost-effectiveness and environmental trade-offs of robotic systems remain insufficiently characterized.

Robotic and minimally invasive approaches occupy a distinct position within the twin transformation framework. While robotic platforms currently function as surgeon-controlled systems rather than autonomous digital agents, they are fundamentally enabled by advanced digital interfaces, high-definition three-dimensional visualization, motion scaling algorithms, and integrated data systems. In this sense, robotic cardiothoracic surgery represents a digitally mediated extension of minimally invasive techniques rather than an independent or fully automated technology. The relevance of this section to digital transformation therefore lies not in surgical access alone, but in the integration of digital visualization, precision engineering, and data-enabled workflow optimization within contemporary operative practice.

From a systems perspective, minimally invasive and robotic techniques may also contribute to reduced material consumption and operational waste. Lower transfusion requirements, decreased use of blood products, and reduced demand for surgical consumables such as sutures, drapes, and dressings have all been reported in association with less invasive approaches. However, the sustainability profile of robotic surgery remains complex. Robotic systems require substantial upfront capital investment, ongoing maintenance, and significant energy consumption, and many robotic instruments rely on disposable components that generate considerable waste. As a result, the long-term environmental impact of widespread robotic adoption remains uncertain, despite clear short-term clinical and patient-centered benefits. Comprehensive life-cycle assessments comparing robotic, conventional minimally invasive, and open surgical techniques are therefore needed to fully evaluate their relative environmental footprints [23].

A broad overview of robotic-assisted technologies in cardiac surgery, invasive cardiology, and even routine hospital tasks has been provided by Koulaouzidis et al., who describe applications ranging from percutaneous coronary interventions and electrophysiology procedures to minimally invasive valve repair, CABG, and structural heart disease interventions. The review highlights several advantages of robotic systems, including enhanced precision, improved maneuverability in complex anatomical settings, reduced radiation exposure for operators, and optimized use of fluoroscopy. At the same time, it underscores key barriers to widespread adoption, such as high installation and instrumentation costs, space requirements within operating rooms, and steep learning curves necessitating specialized training. Despite these challenges, growing acceptance among both clinicians and patients suggests an expanding role for robotic technologies in cardiothoracic care [24].

Clinical experience from specialized centers further illustrates the feasibility and benefits of robotic coronary surgery. Giroletti et al. reported outcomes from fully robotic coronary artery bypass (TE-CAB) and robotic-assisted minimally invasive direct coronary artery bypass (RA-MIDCAB) procedures performed at Humanitas Gavazzeni in Italy. Their work emphasizes the critical role of preoperative imaging, including computed tomography and chest radiography, in guiding port placement, mini-thoracotomy positioning, and left internal mammary artery harvesting. The use of the Da Vinci X system for robotic arm positioning, selective lung ventilation, carbon dioxide insufflation, and optimized patient positioning enabled improved surgical angles, minimized cardiac interference, and enhanced hemodynamic stability, contributing to procedural safety and anastomotic accuracy [25].

Additional support for hybrid robotic approaches is provided by Piperata et al., who described a single-center experience combining robotic harvesting of the left internal mammary artery with minimally invasive direct coronary artery bypass grafting. In this retrospective case series of 17 patients, the procedure was associated with a 100% technical success rate, no 30-day mortality, and relatively short intensive care unit and hospital stays. Despite the small sample size, the findings demonstrate the safety and feasibility of this hybrid strategy and highlight potential advantages, including reduced postoperative pain and fewer pulmonary complications compared with conventional approaches [26].

Robotic innovation in thoracic surgery continues to evolve, as illustrated by the first reported case of fully dual-port robotic-assisted thoracic surgery (F-DRATS) segmentectomy with indocyanine green-guided intersegmental plane identification. Using only two intercostal incisions and avoiding rib spreading, the procedure enabled precise segmental resection with lymphadenectomy and was associated with an uneventful postoperative recovery. This experience emphasizes the potential of advanced robotic techniques to further minimize surgical trauma while maintaining oncological adequacy in complex thoracic procedures [27].

Although minimally invasive surgery can be performed without sophisticated digital infrastructure, its evolution in cardiothoracic practice has been closely intertwined with advancements in imaging, robotic telemanipulation, and perioperative data integration. Within the twin transformation paradigm, the value of these approaches lies in their potential to enhance precision, reduce perioperative morbidity, and improve resource efficiency, thereby aligning clinical innovation with sustainability objectives.

Reported clinical benefits vary across institutions and procedural types, and comparative data between robotic, minimally invasive, and open techniques are not uniformly consistent. Differences in learning curves, institutional expertise, and cost structures further complicate direct comparisons, highlighting the importance of context-specific evaluation.

### 3.3. Digital Twins and Simulation

The emergence of digital twins and advanced simulation technologies is reshaping both preoperative planning and surgical education in cardiothoracic surgery. Digital twin models enable surgeons to rehearse complex procedures in a virtual environment that closely replicates a patient’s anatomy and physiological characteristics prior to entering the operating room. By allowing scenario testing under variable clinical conditions, these technologies enhance surgical precision, improve risk assessment, and support more informed decision-making. Their value is particularly evident in complex valve repairs, congenital heart disease, and anatomically challenging cases, where conventional planning methods may be insufficient to capture patient-specific variability [28,29].

Beyond operative planning, virtual and augmented reality (VR/AR)-based simulation platforms are transforming surgical training. Immersive VR environments facilitate repetitive, skills-based practice in a controlled and resource-efficient setting, increasingly supplementing or replacing traditional reliance on cadaveric dissection and physical models. This shift not only democratizes access to high-quality surgical education but also reduces dependence on finite cadaveric resources and lowers the environmental footprint associated with the production, transport, and disposal of physical training materials. Despite these advantages, challenges remain, including limitations in model accuracy, integration with real-time patient data, and the costs associated with large-scale implementation. Nevertheless, the convergence of digital twin technology with VR/AR simulation represents a significant step toward personalized, digitally enabled, and potentially more sustainable cardiothoracic care [28]. However, most available evidence remains focused on feasibility and technical implementation, with limited prospective data demonstrating measurable improvements in hard clinical outcomes. An unresolved question remains whether widespread adoption of digital twin technologies will meaningfully alter morbidity, mortality, or resource utilization at scale.

Clinical evidence supports the practical impact of digital twins on surgical decision-making. Lippert et al. evaluated the use of mixed reality-based anatomic digital twins for preoperative planning in patients with complex cardiac defects, creating patient-specific models for 50 individuals between September 2020 and December 2022. After initial planning using conventional visualization techniques, multidisciplinary heart teams reassessed each case using the digital twins, resulting in changes to surgical strategy in 68% of patients. Although artificial intelligence accelerated model generation, manual refinement remained necessary in all cases. Importantly, postoperative outcomes supported the safety of this approach, and clinicians reported high satisfaction with the mixed reality experience, emphasizing its value in enhancing anatomical understanding and surgical confidence [29].

The conceptual foundations of digital twin-assisted surgery have been further articulated by Asciak et al., who traced the evolution of digital twin technology from its early application in NASA’s Apollo 13 mission simulators to its widespread adoption in industries such as manufacturing, automotive engineering, and infrastructure management. In healthcare, digital twins are defined by three core components: the physical entity, its virtual representation, and the enabling technologies that allow continuous bidirectional communication between them. This ecosystem is supported by advanced modeling and simulation, artificial intelligence, extended reality visualization, and the Internet of Medical Things, facilitating real-time data exchange and dynamic model updating. Such integration allows physical changes to be rapidly reflected in the virtual model, enabling continuous optimization, prediction, and monitoring throughout the surgical care pathway. Despite their considerable promise, the authors highlight key translational challenges, including data integration, computational demands, and the need for rigorous validation within real-world surgical workflows [30].

However, digital twin research remains largely exploratory, with limited large-scale prospective validation. Model accuracy, data integration challenges, and variability in computational assumptions introduce uncertainty, and real-world clinical impact has yet to be conclusively demonstrated.

### 3.4. Telemedicine and Remote Monitoring

Telemedicine has become an increasingly important adjunct to conventional cardiothoracic care, supported by the expanding use of wearable and implantable monitoring technologies. It enables remote consultations, structured follow-up visits, and longitudinal monitoring of chronic and postoperative conditions, with multiple studies demonstrating its ability to improve access to care for patients in underserved or geographically remote areas while maintaining continuity after surgery. Remote monitoring systems have shown particular promise in the early detection of postoperative complications, such as arrhythmias or wound infections, thereby enabling timely intervention and potentially reducing unplanned readmissions [31,32,33].

From a sustainability perspective, telemedicine contributes directly to reducing healthcare-related carbon emissions by limiting the need for patient travel and associated resource use. These reductions align with broader goals of environmentally responsible healthcare delivery, while also decreasing indirect costs and logistical burdens for patients and healthcare systems. Despite these advantages, equitable access to telehealth remains a significant challenge, particularly for individuals with limited digital literacy, restricted internet connectivity, or socioeconomic barriers. Addressing these disparities requires not only technological solutions but also organizational adaptation, standardized interoperability frameworks, and robust data security protocols [31]. Nevertheless, heterogeneity in telemedicine program design, monitoring intensity, and patient selection limits direct comparison across studies. Long-term data evaluating equivalence or superiority compared with conventional follow-up pathways remain limited.

Clinical evidence supports the effectiveness of structured telemedicine programs in cardiothoracic surgery. Lobdell et al. evaluated the Perfect Care remote monitoring program for patients undergoing adult cardiac surgery, integrating a digital health kit—including a fitness tracker, weighing scale, and sphygmomanometer—with a dedicated application for biometric monitoring, scheduling, audiovisual visits, and secure messaging. Compared with propensity-matched controls undergoing coronary artery bypass, valve, or combined procedures, program participants experienced shorter postoperative hospital stays, lower 30-day readmission and mortality rates, and reduced outcome variability. These findings suggest that structured telemedicine-based postoperative care can enhance recovery while reducing healthcare utilization, particularly when individualized biometric monitoring is combined with strategies to address access disparities [32].

Complementary findings were reported in a larger retrospective analysis by the same group, which compared 1000 adult cardiac surgery patients enrolled in a remote perioperative monitoring program with 1000 propensity-matched controls. The program incorporated biosensors, patient-reported outcomes, audiovisual visits, and secure messaging. Patients in the remote monitoring cohort were more frequently discharged directly home, had a shorter median postoperative length of stay, and experienced a 33% relative reduction in 30-day readmissions. Together, these results demonstrate the feasibility and clinical benefit of remote perioperative monitoring in improving postoperative outcomes and optimizing resource use in cardiothoracic surgery [34].

Beyond direct patient care, telemedicine platforms are also reshaping surgical education and professional collaboration. Hudspeth et al. described hybrid thoracic surgery observerships at the Mayo Clinic that combined virtual and in-person participation using a remote presence platform. Through real-time control of multiple camera views, students were able to observe live operations and telemedicine consultations, interact with surgical teams, and engage in educational discussions without the need for continuous physical presence. Compared with traditional in-person observerships, this hybrid model enhanced accessibility, particularly for students facing geographic or financial constraints, while offering new opportunities for training, teamwork, and continuing medical education [33].

### 3.5. Electronic Health Records (EHRs) and Data Interoperability

Electronic Health Records (EHRs) constitute the foundational infrastructure of digital transformation in modern healthcare, providing centralized platforms for the storage, retrieval, and continuous updating of patient data. In cardiothoracic surgery, EHRs support integrated, patient-centered care by enabling seamless information exchange among multidisciplinary teams, including surgeons, anesthesiologists, cardiologists, intensivists, and nursing staff, across all phases of the patient journey. When effectively implemented, EHR systems enhance continuity of perioperative care, reduce redundant diagnostic testing, and improve coordination between clinical services. Beyond their immediate clinical utility, EHRs facilitate longitudinal outcome monitoring, quality improvement initiatives, and data-driven benchmarking across institutions [35].

The aggregation of structured and unstructured patient data within EHRs also enables advanced analytics and machine learning applications. Large-scale datasets allow for the early identification of complications, detection of inequities in care delivery, and evaluation of best practices, thereby supporting personalized medicine and multicenter research collaboration. From a systems perspective, these capabilities contribute to more efficient resource utilization by informing clinical decision-making, reducing unnecessary interventions, and supporting predictive modeling across the perioperative continuum [35,36].

Despite their transformative potential, significant barriers to optimal EHR use persist. Interoperability challenges, fragmented data standards, and persistent data silos often limit effective information exchange between systems and institutions. Usability concerns and increased administrative workload can further hinder clinician engagement, while issues related to data privacy, cybersecurity, and regulatory compliance require ongoing attention. Addressing these challenges through improved interoperability standards, user-centered system design, and streamlined documentation workflows is essential for realizing both the clinical and sustainability objectives of contemporary cardiothoracic surgery [36].

Practical examples illustrate how interoperable EHR infrastructures can support advanced predictive analytics in cardiothoracic care. The Healthcare Enabled by Artificial Intelligence in Real Time (HEART) project described by Mazhude et al. represents a multidisciplinary collaboration between clinicians, data scientists, and industry partners aimed at developing real-time predictive models for cardiothoracic intensive care units. By integrating static and dynamic preoperative, intraoperative, and postoperative variables derived directly from EHRs, the project seeks to forecast adverse outcomes such as acute kidney injury, atrial fibrillation, stroke, and prolonged hospital stay. The establishment of a validated data collection pipeline and the development of clinician-friendly interfaces highlight the potential of EHR-integrated analytics to support timely decision-making, improve patient outcomes, and reduce postoperative complications [37].

Similarly, Weiss et al. demonstrated the value of institution-specific, multimodal EHR data for individualized risk prediction in cardiac surgery. By analyzing 4016 clinical features from 6392 patients, the authors developed an Extreme Gradient Boosting model that outperformed conventional Society of Thoracic Surgeons risk scores across multiple performance metrics, including F-measure and area under the curve. These findings suggest that combining population-based risk models with institution-specific EHR-driven analytics can enhance patient-level decision-making and improve predictive accuracy, reinforcing the central role of interoperable, high-quality EHR systems in enabling advanced digital health applications [38]. An overview of the key digital technologies currently shaping cardiothoracic surgery, together with their principal clinical applications and reported benefits across the perioperative pathway, is provided in Table 1 [39,40,41,42,43,44,45,46,47,48,49,50,51,52,53,54,55,56,57,58,59,60,61,62,63,64,65,66,67,68,69,70,71,72,73].

### 3.6. Congenital and Pediatric Cardiac Surgery

Congenital and pediatric cardiac surgery represents a central pillar of the cardiothoracic specialty and a key element of the broader cardiothoracic triad. Congenital heart disease (CHD) is the most common birth defect worldwide, with many affected individuals requiring surgical intervention early in life and ongoing multidisciplinary care involving pediatric cardiologists, intensivists, anesthesiologists, radiologists, and allied specialists [39,40]. Survival into adulthood has improved markedly in recent decades owing to advances in surgical and perioperative management, underscoring the lifelong commitment to care that this patient population requires [39].

Digital transformation plays a particularly important role in congenital and pediatric cardiac surgery. Advanced imaging modalities such as high-resolution computed tomography (CT), magnetic resonance imaging (MRI), and three-dimensional (3D) modeling facilitate detailed anatomical assessment and preoperative planning in complex heart defects [41,42]. Innovations such as 3D virtual and printed heart models have been demonstrated to enhance surgical strategy, improve team communication, and support education and training across multidisciplinary teams [41,42]. Augmented and virtual reality applications are also emerging, enabling immersive visualization that may further refine procedural planning and risk stratification [41,43].

From a sustainability perspective, congenital cardiac surgery presents unique clinical and ethical considerations. Procedures are frequently intricate and resource-intensive, but early and effective surgical intervention can lead to decades of improved survival, quality of life, and reduced long-term morbidity [39,40]. Additionally, digital tools such as telemedicine and remote follow-up models hold promise for optimizing longitudinal care for pediatric CHD patients, especially those in geographically underserved regions, thereby aligning with principles of equitable and sustainable healthcare delivery [43,44].

## 4. Sustainability in Cardiothoracic Surgery

### 4.1. Environmental Burden of Operating Rooms

Hospital operating rooms represent some of the most resource-intensive environments in modern healthcare, and cardiothoracic surgery imposes a particularly heavy environmental burden due to its reliance on energy-intensive equipment, complex life-support systems, anesthetic gases, and large volumes of disposable materials. Advanced surgical instruments, continuous high-efficiency ventilation and air conditioning (HVAC) systems, and stringent lighting requirements necessary for sterility and visibility are major contributors to operating room energy consumption. Collectively, these factors significantly increase the carbon footprint of surgical care and, by extension, the healthcare sector as a whole [45,46,47,48].

Anesthetic gases constitute a major and often underrecognized source of greenhouse gas emissions. Agents such as nitrous oxide and desflurane possess global warming potentials many times greater than that of carbon dioxide. Given the length and complexity of cardiothoracic procedures, these agents are often used in larger quantities, amplifying their environmental impact. In parallel, the extensive use of single-use surgical instruments, drapes, gowns, tubing, and extracorporeal circulation components generates substantial waste streams. Although disposable materials play a critical role in infection prevention and patient safety, their production, transport, and disposal—often through energy-intensive incineration—carry significant environmental costs [45].

Recent life cycle assessment studies indicate that cardiothoracic surgery ranks among the surgical specialties with the highest per-procedure carbon footprints. Addressing this burden requires targeted sustainability strategies, including comprehensive assessment of material life cycles, adoption of lower-impact anesthetic practices, and optimization of operating room energy use [46]. Quantifying environmental impact at the procedural level has therefore emerged as a key step toward evidence-based sustainability interventions.

Blitzer et al. highlighted the substantial greenhouse gas emissions associated with cardiothoracic surgery, reporting per-case emissions ranging from 124 kg to 505 kg CO_2_ equivalents based on life cycle assessments conducted in the United States and France. Cardiopulmonary bypass circuits and disposable materials—particularly plastic components—were identified as the primary contributors. The authors also noted a relative lack of detailed emissions data for thoracic surgical procedures and demonstrated that less invasive transcatheter interventions, such as catheter ablation for atrial fibrillation, are associated with markedly lower emissions, estimated at approximately 77 kg CO_2_ equivalents per procedure. These findings emphasize the need for further research aimed at measuring, comparing, and ultimately reducing the environmental footprint of cardiothoracic interventions [47].

Complementary evidence is provided by van Bree et al., who conducted a life cycle assessment of elective coronary artery bypass grafting in a Dutch academic hospital. A single patient trajectory was associated with approximately 414 kg CO_2_ equivalents, with major contributions arising from energy consumption, staff commuting, and disposable operating room supplies. Extracorporeal circulation sets, surgical drapes, intraoperative cell salvage systems, gowns, and gauzes were the dominant sources of material-related emissions, while HVAC systems accounted for the majority of energy use. The authors proposed several mitigation strategies, including minimizing unnecessary disposables, substituting reusable alternatives where feasible, improving operating room energy efficiency, expanding the use of renewable energy sources, and promoting low-emission commuting practices among healthcare staff [48].

Beyond quantification, broader frameworks have been proposed to guide sustainable practice in cardiothoracic surgery. Leow et al. emphasized the role of the specialty in mitigating health risks associated with climate change, advocating for the formal integration of sustainability principles into surgical education, routine carbon auditing, and life cycle analysis. They highlighted pragmatic interventions such as reducing excess equipment use, favoring reusable over single-use materials when clinically appropriate, and coordinating resources across surgical services. The authors endorsed the “Triple Bottom Line” framework, which balances environmental responsibility, economic viability, and quality of care, as a guiding principle for sustainable surgical practice [49].

At a broader cardiovascular care level, Rajagopalan et al. reviewed the environmental impact of healthcare delivery and outlined mitigation frameworks including carbon accounting, waste prevention, life cycle assessment, and circular economy models. Their analysis emphasized prevention as a powerful strategy for reducing environmental impact and discussed the practical challenges of integrating sustainability into routine clinical care without compromising patient outcomes or societal benefit. Together, these frameworks provide a strategic foundation for embedding sustainability into cardiothoracic surgery while maintaining high standards of clinical excellence [50].

### 4.2. Green Surgical Practices

Sustainable surgical practices aim to reduce the environmental impact of cardiothoracic procedures while preserving patient safety and procedural effectiveness. One of the most prominent areas of focus is the choice between reusable and disposable surgical instruments. Although single-use products offer convenience and guaranteed sterility, their manufacture, transportation, and disposal are energy-intensive and generate substantial waste. In contrast, high-quality reusable instruments, when appropriately sterilized and maintained, can significantly reduce material consumption and associated emissions. However, their adoption requires careful consideration of initial procurement costs, sterilization capacity, and logistical workflows to ensure clinical safety and economic feasibility [51,52,53].

Beyond instrument selection, waste reduction and recycling initiatives are increasingly being implemented within cardiothoracic operating rooms. Practical measures include minimizing excessive packaging, segregating waste streams, and recycling eligible materials, all of which can substantially decrease the volume of waste sent to landfills. In parallel, several institutions have adopted lean operating room workflows by optimizing instrument trays, eliminating unnecessary disposables, and streamlining surgical setup processes. These approaches not only advance sustainability goals but also improve operational efficiency by reducing setup times, storage requirements, and overall material handling, demonstrating that environmental responsibility can be integrated into routine surgical practice without compromising patient outcomes [51].

Empirical evidence highlights the scale and structure of waste generation in cardiovascular procedures. Amin et al. quantified waste produced during cardiac operations and catheterization laboratory interventions, demonstrating that complex procedures—such as structural heart interventions and chronic total occlusion percutaneous coronary interventions—generate particularly high volumes of waste. Importantly, the authors noted that a substantial proportion of this waste is potentially recyclable or avoidable, underscoring the opportunity for targeted waste diversion strategies. To support global net-zero emission targets, they advocated for comprehensive sustainability initiatives encompassing waste reduction, reuse, recycling, and closer engagement with supply chains, energy consumption, and material management practices across healthcare systems [52].

A broader perspective on sustainable cardiovascular care is provided by Barratt et al., who reviewed carbon dioxide equivalent emissions across multiple domains, including diagnostic imaging, pacemaker monitoring, pharmacotherapy, and in-hospital surgical care. Their analysis revealed marked variability in environmental impact depending on modality, with echocardiography associated with substantially lower emissions than cardiac magnetic resonance imaging or single-photon emission computed tomography. The review identified several feasible mitigation strategies, such as prioritizing low-emission diagnostic tools, expanding telemonitoring, and adopting efficient surgical techniques—including optimized management of cardiopulmonary bypass circuitry. Collectively, these measures illustrate how environmentally conscious clinical choices can reduce emissions while delivering co-benefits for patient care, healthcare costs, and societal well-being [53].

### 4.3. Hospital-Level Sustainability Initiatives

Beyond individual surgical procedures, hospital-wide sustainability initiatives play a decisive role in reducing the environmental footprint of cardiothoracic surgery. Institutional programs targeting energy efficiency, procurement practices, and organizational standards can substantially mitigate the environmental impact of surgical care. Investments in energy-efficient infrastructure—such as advanced climate control systems, LED lighting, and optimized heating, ventilation, and air conditioning (HVAC) systems—have been shown to reduce greenhouse gas emissions while simultaneously lowering operational costs. Addressing building-level energy consumption is therefore a critical component of sustainable perioperative care [54,55].

Sustainable procurement represents a second major pillar of hospital-level environmental responsibility. By prioritizing reusable or recyclable medical devices, products with reduced life-cycle emissions, and suppliers that employ environmentally responsible packaging and manufacturing practices, hospitals can embed sustainability considerations throughout the supply chain. Achieving an appropriate balance between cost, quality, and environmental impact requires close collaboration among surgical teams, procurement departments, and hospital administration, ensuring that sustainability goals align with clinical safety and economic constraints [49,54].

Formal governance frameworks and certification schemes further support the implementation and evaluation of sustainable practices. Standards such as Leadership in Energy and Environmental Design (LEED), ISO 14001 environmental management systems, and healthcare-specific sustainability accreditations provide structured approaches for benchmarking performance, promoting continuous improvement, and demonstrating institutional commitment to environmental stewardship. Adoption of these frameworks fosters a culture that supports sustainable cardiothoracic surgery by encouraging both individual accountability and coordinated organizational action to minimize resource use and ecological impact [54].

As one of the most resource-intensive medical specialties, cardiothoracic surgery carries a particular responsibility to address healthcare-related carbon emissions both within and beyond the operating room. This responsibility positions cardiothoracic surgeons as potential leaders in environmental stewardship. Several authors argue that sustainability should be embedded within surgical culture, beginning with its formal inclusion in training curricula and reinforced through routine use of tools such as life cycle assessments and carbon audits. Pragmatic interventions—including reducing unnecessary equipment use, transitioning from disposable to reusable instruments when clinically appropriate, and avoiding low-value diagnostic testing through judicious clinical decision-making—have been identified as feasible and impactful measures [49,55].

Broader systemic approaches, such as resource sharing across departments and institutions, further align with the principles of the Triple Bottom Line framework, which seeks to balance clinical outcomes, financial responsibility, and environmental impact [49,56]. Collectively, these strategies support a paradigm shift in surgical practice, reframing sustainability as a core component of high-quality care rather than an ancillary consideration. Given its technological sophistication and resource intensity, cardiothoracic surgery is uniquely positioned to lead healthcare decarbonization efforts and to establish benchmarks for environmentally responsible perioperative practice, procurement, and institutional accountability.

### 4.4. Clinical and Ethical Rationale

Beyond environmental considerations, sustainability in cardiothoracic surgery has direct and meaningful implications for patient care. Measures aimed at reducing energy consumption, minimizing waste, and selecting lower-impact anesthetic techniques can contribute to safer, cleaner, and more efficient surgical environments, indirectly supporting improved clinical outcomes. Leaner workflows and reduced reliance on disposable materials may simplify operative processes, decrease clutter, and lower the risk of errors or postoperative infections. At the same time, patients are increasingly aware of and receptive to environmentally responsible healthcare practices, aligning sustainability initiatives with patient-centered care principles and broader societal expectations [57].

From an ethical perspective, healthcare systems face a dual responsibility: to provide effective, high-quality care for individual patients while simultaneously minimizing harm to population health and the environment. Climate change poses a substantial threat to global health, and the healthcare sector itself is a significant contributor to greenhouse gas emissions. Cardiothoracic surgery exemplifies this tension, given its high resource intensity and technological demands. By integrating sustainability into clinical decision-making, hospitals and surgical teams fulfill an ethical obligation to reduce environmental harm, conserve finite resources, and protect both current and future patient populations. When sustainability is framed as an integral component of clinical ethics, it reinforces the alignment between patient care, technological innovation, and societal responsibility rather than positioning environmental considerations as competing priorities [58].

The ethical scope of sustainability in cardiothoracic surgery extends beyond individual institutions to encompass global health equity. Cardiovascular disease remains a leading cause of morbidity and mortality worldwide, yet access to timely, safe, and affordable cardiac surgery is profoundly unequal. Nearly six billion people lack adequate access to essential surgical care, and despite this unmet need, cardiac surgery has historically received limited attention within global health and policy agendas. In this context, recent work has explored the relationship between cardiac surgery and the United Nations’ 17 Sustainable Development Goals (SDGs), demonstrating that at least 15 of these goals are directly linked to the provision of cardiac surgical care. While SDG 3 (“Good Health and Well-Being”) is the most immediately relevant, cardiac surgery also intersects with broader goals related to gender equality, education, infrastructure development, poverty reduction, and innovation [59].

This broader perspective reframes cardiothoracic surgery not merely as a specialized and resource-intensive medical service, but as a socio-economic determinant of global development and health equity. The authors advocate for a more comprehensive ethical framework that integrates sustainability, human rights, and global justice into surgical practice. Key priorities include interdisciplinary collaboration, prevention-oriented public health strategies, and more equitable clinical research that includes low- and middle-income populations frequently underrepresented in current studies. Within this framework, cardiothoracic surgeons are encouraged to act not only as clinicians but also as advocates—engaging with policymakers, fostering international partnerships, and promoting the recognition of cardiac surgery as an essential component of universal health coverage.

Importantly, environmental impact assessments are methodologically heterogeneous, often relying on life-cycle modeling assumptions that vary between institutions and healthcare systems. Comparative carbon footprint data across procedures remain incomplete, and standardized measurement frameworks are still evolving.

An overview of key sustainability challenges in cardiothoracic surgery and evidence-based mitigation strategies at both the operating room and institutional levels is provided in Table 2.

## 5. The Synergy of Twin Transformation in Cardiothoracic Surgery

### 5.1. Digital Tools Driving Sustainability

The preceding sections intentionally outlined the digital and sustainability domains separately. However, the central contribution of this review lies not in cataloging technologies or environmental challenges in isolation, but in examining their structural convergence under the twin transformation paradigm. The following section therefore synthesizes these domains, highlighting how digital innovation and sustainability operate as mutually reinforcing strategic vectors within cardiothoracic surgery.

Although the conceptual synergy between digital transformation and sustainability is compelling, empirical data directly measuring their combined impact remain limited. Most available studies examine digital or environmental interventions separately, leaving the magnitude of their interactive effect insufficiently quantified.

Digital technologies increasingly enable cardiothoracic surgery to align clinical innovation with environmental sustainability, demonstrating how digital transformation can directly reduce resource consumption while maintaining high-quality patient care. One prominent area of impact is operating room and supply chain optimization. Artificial intelligence-driven scheduling systems can minimize idle operating room time, optimize staff allocation, and more accurately predict procedure duration and equipment requirements. By preventing unnecessary instrument preparation, repeated sterilization cycles, and inefficient resource use, these tools contribute to lower energy consumption and reduced operational costs, illustrating how efficiency gains translate into tangible sustainability benefits.

Telemedicine further exemplifies the synergistic relationship between digital innovation and environmental responsibility. Remote consultations, postoperative follow-up, and long-term monitoring reduce the need for patient travel, particularly for individuals residing far from tertiary cardiothoracic centers. These reductions in transportation-related emissions align directly with climate-conscious healthcare objectives, while simultaneously supporting continuity of care, early complication detection, and patient convenience. In this way, telehealth achieves parallel clinical and environmental gains rather than forcing trade-offs between them [60].

Digital training technologies represent another important mechanism through which sustainability is advanced. Virtual reality (VR)-based simulation reduces reliance on cadavers, physical models, and disposable training materials by providing immersive, repeatable learning environments. Surgical teams can practice complex procedures under controlled conditions, improving technical proficiency and patient safety while conserving physical resources. This approach exemplifies the core principle of twin transformation: technology-driven performance enhancement that simultaneously supports environmental stewardship [61].

The sustainability benefits of digital education are particularly evident in low-resource settings. Anyinkeng et al. described how tele-mentoring systems, augmented and virtual reality platforms, and AI-assisted training tools are transforming cardiothoracic surgical education in environments where traditional apprenticeship models are often impractical. These scalable digital solutions enable standardized skills assessment, real-time expert supervision, and risk-free procedural rehearsal across geographic boundaries. Evidence from African implementation initiatives—including tele-mentoring programs in Rwanda—demonstrates improved surgical outcomes without compromising patient safety. Nonetheless, persistent barriers such as limited internet infrastructure, high implementation costs, and resistance to shifting away from established training paradigms remain significant. The authors advocate a multipronged strategy involving integration of digital platforms into national training curricula, investment in affordable simulation technologies, international partnerships, and adaptation of instructional models to local constraints. Successful initiatives, such as the Pan-African Association of Surgeons’ virtual reality programs and Operation Smile’s remote mentorship networks, highlight the feasibility of this approach [62].

Extended reality (XR) technologies—including virtual, augmented, and mixed reality—further expand the potential of sustainable, simulation-based training in cardiothoracic surgery. As duty-hour restrictions and patient safety imperatives have limited traditional apprenticeship opportunities, XR platforms now provide high-fidelity, repeatable simulations of complex procedures such as robotic-assisted interventions, valve repair, coronary artery bypass grafting, and thoracic resections. These systems support remote mentoring, preoperative rehearsal, and exposure to rare or high-risk scenarios without jeopardizing patient safety. However, challenges remain, including limited haptic realism, high development costs, and restricted access in low-resource settings. Accurately reproducing complex thoracic anatomy and dynamic cardiac physiology also remains technically demanding, raising concerns about overreliance on virtual environments with incomplete real-world fidelity. Future directions include the development of more affordable XR platforms, enhanced tactile feedback systems, AI-driven adaptive learning, and competency-based assessment frameworks. Collectively, these advances position XR technologies as powerful enablers of globally scalable, resource-efficient cardiothoracic training [63].

### 5.2. Sustainability Stimulating Digital Innovation

Sustainability and environmental objectives are increasingly acting as catalysts for digital innovation in cardiothoracic surgery. Hospitals and healthcare systems facing pressure to reduce carbon emissions and improve resource efficiency are adopting digital solutions not only for clinical optimization but also to meet institutional and regulatory sustainability targets. Telehealth platforms, artificial intelligence-driven logistics, and digital twin technologies are being implemented as strategic responses to environmental constraints, illustrating how sustainability imperatives can actively stimulate technological advancement rather than hinder it [64].

Artificial intelligence plays a central role in this transition by enabling more efficient management of surgical inventories and supply chains. AI-driven systems can forecast procedural requirements, optimize stock levels, and reduce over-preparation of instruments and consumables, thereby minimizing waste and unnecessary sterilization cycles. Similarly, the rapid expansion of telehealth and remote patient monitoring has been partly driven by sustainability goals, as these technologies significantly reduce emissions associated with patient and caregiver travel while maintaining high standards of postoperative care and clinical surveillance [64]. In this way, environmental responsibility directly incentivizes the adoption of digital care models.

Digital twin technologies further exemplify this feedback loop between sustainability and innovation. By enabling in silico testing of patient-specific interventions, digital twins reduce reliance on resource-intensive trial-and-error approaches in clinical practice. Virtual simulation of surgical strategies and disease trajectories allows clinicians to anticipate complications, refine treatment plans, and optimize perioperative decision-making before physical intervention occurs. These capabilities support more precise, resource-efficient care delivery while reinforcing the value proposition of digital investment from both clinical and environmental perspectives [64,65].

The sustainability-driven innovation potential of digital twins is explored in depth by Sel et al., who describe digital twins as continuously updated virtual representations of individual cardiovascular systems, capable of integrating complex data streams from imaging, physiological monitoring, genomics, and wearable sensors. Using artificial intelligence, mechanistic modeling, and sensor-enabled bidirectional feedback loops, these models can generate real-time predictions, simulate disease progression, and support long-term treatment optimization. Importantly, the authors emphasize the engineering and governance frameworks required for clinical deployment, including rigorous validation, verification, and uncertainty quantification to ensure clinician trust and patient safety. While challenges related to computational scalability, data standardization, and ethical oversight remain, digital twins are positioned as a foundational technology for future cardiovascular care that is not only more precise but also more sustainable. By reducing unnecessary procedures, inefficient resource use, and avoidable interventions, digital twins illustrate how environmental responsibility can directly drive the development of advanced digital healthcare solutions [65].

### 5.3. Case Studies/Pilot Projects

Early evidence of the synergistic benefits of twin transformation is emerging from cardiothoracic centers that have begun integrating digital innovation with sustainability-oriented strategies. In several high-volume cardiac surgery units, the combination of telemedicine-based follow-up with AI-driven operating room scheduling has been associated with reduced operational delays, improved staff allocation, and lower emissions related to patient travel. Similarly, institutions implementing reusable instrument programs alongside virtual reality-based surgical training have reported measurable reductions in disposable waste without compromising surgical competency or educational outcomes. Preliminary assessments also suggest potential cost-effectiveness benefits arising from decreased material consumption and more efficient use of resources [66,67,68].

These pilot initiatives highlight how digitally enabled workflow optimization and predictive analytics can indirectly support sustainability by preventing complications that would otherwise necessitate additional interventions, prolonged hospital stays, or readmissions. Optimized perioperative pathways contribute to reduced material use, lower energy consumption, and decreased staff overtime. Although most available evidence is derived from single-center experiences or early-stage pilot programs, the consistency of reported clinical, operational, and environmental benefits supports the premise that coordinated digital and sustainability strategies can generate tangible value. Nevertheless, multicenter studies and standardized evaluation frameworks are needed to validate these findings and establish scalable models for broader implementation [66].

Beyond institutional pilots, enabling technologies have been examined through focused reviews that provide insight into their practical applicability. Sadeghi et al. reviewed the use of extended reality (XR)—encompassing virtual, augmented, and mixed reality—in cardiothoracic surgery, identifying applications across preoperative planning, intraoperative guidance, postoperative management, rehabilitation, and patient education. Their nonsystematic review demonstrated growing evidence for the feasibility and utility of XR technologies in surgical simulation, navigation, pain management, and televirtuality. While the authors emphasized the expanding scope of XR applications, they also noted the need for further technological refinement and clinical validation prior to widespread adoption, particularly in routine surgical workflows [67].

Large-scale population-based digital twin projects further illustrate the long-term potential of sustainability-driven digital innovation. Qian et al. developed extensive cardiac digital twin (CDT) populations using cardiac magnetic resonance imaging and electrocardiographic data, generating 3461 models from the UK Biobank and an additional 359 from a cohort with ischemic heart disease. These physics- and physiology-constrained models enabled personalized in silico analysis of electrophysiological behavior across populations, revealing mechanistic insights that are often inaccessible through conventional diagnostic approaches. Notably, the study demonstrated sex-specific differences in QRS duration driven by myocardial anatomy, age- and obesity-related variation in myocardial conduction velocity, and altered repolarization dynamics associated with obesity in female patients. Importantly, several electrophysiological parameters were also linked to lifestyle factors, mental health indicators, and adverse clinical outcomes, underscoring the capacity of digital twin populations to map disease risk and physiological variability at scale [68].

**Figure 2:**
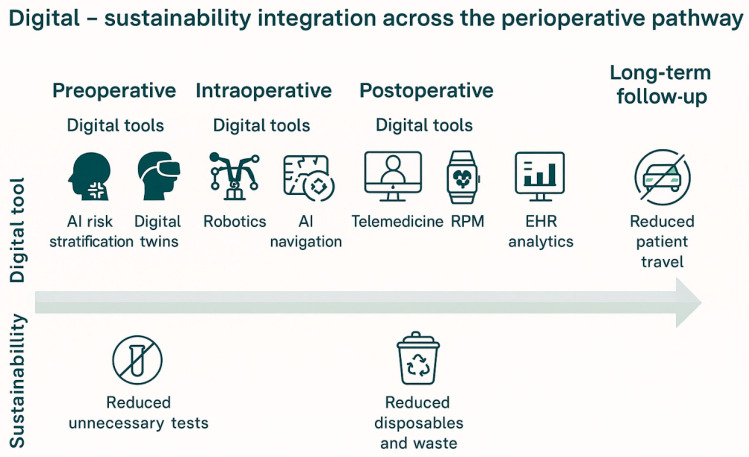
Integration of digital technologies and sustainability strategies across the perioperative pathway in cardiothoracic surgery. Digital tools applied at each phase of care—from preoperative planning to long-term follow-up—contribute to improved clinical outcomes while simultaneously reducing unnecessary testing, disposable use, patient travel, and overall resource consumption, illustrating the operational dimension of the twin transformation framework.

Together, these examples illustrate different levels at which twin transformation can operate—from localized pilot projects and institutional workflow redesign to population-scale modeling initiatives. While the maturity and scope of these efforts vary, they collectively demonstrate how digital technologies can support sustainability objectives by enhancing efficiency, reducing waste, and enabling more precise, preventive, and resource-conscious cardiothoracic care. The bidirectional synergy between digital technologies and sustainability goals in cardiothoracic surgery, which underpins the twin transformation paradigm, is illustrated in Table 3. Figure 2 depicts how digital tools and sustainability objectives interact across the preoperative, intraoperative, postoperative, and long-term follow-up phases of cardiothoracic care.

## 6. Challenges and Barriers

The implementation of twin transformation in cardiothoracic surgery is accompanied by substantial financial, technical, organizational, and regulatory challenges. One of the most prominent barriers is the high upfront investment required for both digital and sustainability-oriented technologies. Capital-intensive initiatives—including energy-efficient operating rooms, reusable instrument systems, advanced waste management infrastructure, robotic surgical platforms, artificial intelligence solutions, and digital twin modeling tools—often demand significant initial funding. Although these investments may yield long-term cost savings through improved efficiency, reduced resource consumption, and lower complication rates, the financial burden can be prohibitive, particularly for institutions in low- and middle-income settings [69].

Technical challenges further complicate implementation. Effective integration of diverse digital systems—such as telemedicine platforms, surgical robotics, electronic health records, artificial intelligence algorithms, and digital twin environments—requires robust interoperability, standardized data architectures, and continuous system maintenance. Ensuring reliable data management across these platforms is essential for clinical accuracy and operational efficiency. At the same time, increased digital connectivity heightens vulnerability to cybersecurity threats, making data protection and privacy safeguards a critical concern. Any breach or system failure has the potential to undermine patient trust and compromise sensitive clinical information [70]. Moreover, the clinical reliability of AI-driven models, virtual reality simulations, and predictive analytics is paramount; insufficient accuracy, bias, or lack of validation may pose risks to patient safety, underscoring the need for rigorous testing, transparent algorithms, and ongoing performance monitoring.

Beyond financial and technical constraints, organizational and cultural factors often represent significant barriers to adoption. Resistance to change among surgical teams may arise from concerns regarding workflow disruption, increased workload, or skepticism toward unfamiliar technologies. Established professional hierarchies and deeply ingrained clinical routines can further slow implementation. Successful change management therefore requires strong leadership commitment, targeted education and training, and clear demonstration of both clinical and operational benefits. Aligning multidisciplinary teams around shared sustainability and digital goals necessitates careful consideration of existing workflows, role responsibilities, and perceived impacts on daily practice [71].

**Figure 3:**
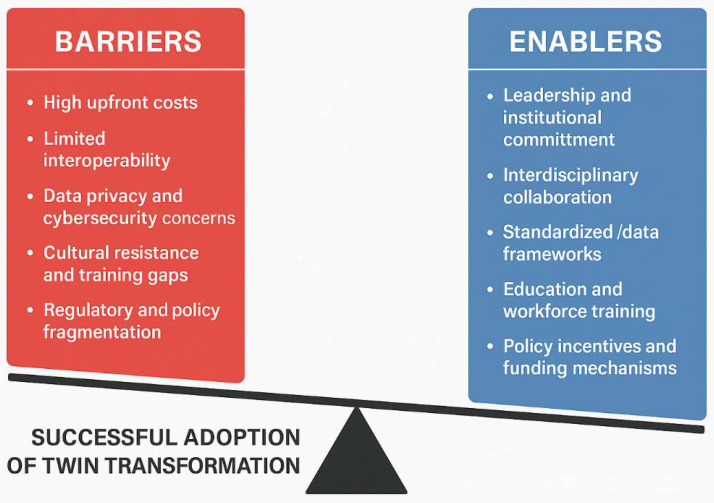
Key barriers and enabling factors influencing the implementation of twin transformation in cardiothoracic surgery. Successful adoption requires alignment of technological, organizational, economic, and regulatory dimensions.

Finally, regulatory and policy frameworks frequently lag behind technological and sustainability advancements. Standards governing the safe, ethical, and effective use of artificial intelligence, telemedicine, and digital twin technologies in surgery remain fragmented and inconsistently applied across regions. Similarly, there is a lack of harmonized international guidelines specifically addressing environmentally sustainable surgical practice. Healthcare institutions are often required to balance innovation with regulatory compliance, while policymakers face the challenge of developing frameworks that protect patients without stifling technological progress. Addressing these regulatory gaps is essential for scaling twin transformation initiatives across diverse healthcare systems and ensuring equitable access to sustainable, digitally enabled cardiothoracic care [72]. The main barriers to implementation and the key enabling factors required for the successful adoption of the twin transformation framework in cardiothoracic surgery are summarized in Figure 3.

## 7. Discussion and Future Directions

The twin transformation paradigm—anchoring the integration of digital innovation and sustainability in cardiothoracic surgery—represents a promising yet still emerging approach to reconciling clinical excellence with ecological responsibility. While the preceding sections highlight substantial progress, the translation of this framework into routine practice requires coordinated advances in metrics, technology, governance, and evidence generation.

### 7.1. Toward Integrated Clinical and Environmental Performance Metrics

A critical next step in advancing twin transformation is the development of standardized metrics that integrate clinical outcomes with environmental impact, enabling objective assessment of sustainability performance alongside patient-centered benefits. Such composite indicators could facilitate cross-institutional benchmarking and support evidence-based prioritization of interventions that simultaneously improve care quality and reduce resource consumption [73]. By quantifying the ecological footprint of cardiothoracic procedures in parallel with traditional surgical outcomes, healthcare systems may better align quality improvement initiatives with sustainability goals.

Recent work in healthcare sustainability has emphasized the importance of carbon accounting and life-cycle assessment frameworks to quantify emissions across clinical pathways and compare alternative models of care, including telemedicine-based follow-up strategies [74,75,76,77,78]. Incorporating these approaches into cardiothoracic surgery could support value-based decision-making and foster transparency in sustainability performance reporting.

### 7.2. AI-Driven Operational Efficiency as a Sustainability Lever

Artificial intelligence-driven logistics and predictive analytics represent a key convergence point between digital innovation and sustainability. Predictive algorithms can optimize operating room utilization, supply chain management, and patient flow, reducing idle time, excess inventory, unnecessary sterilization cycles, and procedural delays. These efficiency gains translate into lower energy consumption, reduced waste generation, and decreased operational costs, while maintaining high standards of patient safety and care quality [73].

At the same time, emerging literature highlights the need to critically evaluate the environmental footprint of digital technologies themselves. AI model training, data storage, and computational demands can be energy-intensive, underscoring the importance of adopting energy-efficient algorithms and sustainable computing strategies to ensure that digital solutions deliver net environmental benefits [79,80].

### 7.3. Digital Twins and the Future of Personalized, Resource-Efficient Surgery

Digital twin technology is poised to redefine personalized surgical planning by enabling patient-specific simulation of anatomical and physiological responses. By replicating individual patient characteristics in silico, digital twins allow clinicians to optimize procedural strategies, reduce intraoperative uncertainty, and minimize complication risk. Future research should prioritize the integration of real-time patient data streams with artificial intelligence analytics to develop adaptive digital twin platforms capable of guiding intraoperative decisions and resource allocation dynamically [81].

At present, most evidence supporting digital twin applications in cardiothoracic surgery is derived from pilot studies or single-center experiences. Large-scale validation efforts and multicenter collaborations will be essential to confirm clinical effectiveness, economic feasibility, and sustainability benefits across diverse healthcare settings [75]. Population-level digital twin initiatives further suggest the potential for risk stratification and disease modeling at scale, opening new avenues for preventive and resource-conscious cardiovascular care [82].

### 7.4. Strengthening the Evidence Base Through Multicenter Research

The predominance of single-center and retrospective studies represents a key limitation in the current evidence base for twin transformation. To establish generalizable best practices, prospective multicenter trials are needed to evaluate combined digital and sustainability interventions using standardized clinical, economic, and environmental endpoints. Such studies could clarify which strategies deliver the greatest impact across varied institutional contexts and resource settings [83].

Recent systematic reviews in surgical sustainability and digital health consistently identify heterogeneity in study design, outcome reporting, and methodological rigor as barriers to translation, reinforcing the urgency of coordinated research frameworks and shared evaluation standards [84].

### 7.5. Policy, Funding, and Governance as Enablers of Scale-Up

The successful transition of twin transformation from pilot initiatives to standard practice will depend heavily on supportive policy environments and financial incentives. Governments, health systems, and regulatory bodies can play a decisive role by establishing guidelines for environmentally responsible surgical practice, funding digital and sustainable infrastructure, and incentivizing institutions that demonstrably reduce emissions while maintaining high-quality care [73].

Clear regulatory guidance for artificial intelligence, telemedicine, digital twins, and extended reality technologies is also essential to balance innovation with patient safety and ethical accountability. Recent policy-oriented analyses emphasize the need for governance frameworks that explicitly incorporate environmental considerations into digital health regulation [85,86].

### 7.6. Ethical, Equity, and Global Health Implications

Cardiothoracic surgery stands at a unique ethical crossroads, given its high resource intensity and global disparities in access to care. Sustainability in this context is inseparable from health equity, as climate change disproportionately affects vulnerable populations and threatens long-term public health. Embedding sustainability within clinical ethics reinforces the responsibility of surgical systems to protect both current and future patients while minimizing harm to population health [87].

Recent scholarship demonstrates that environmentally sustainable healthcare practices can also support social justice by reducing inefficiencies and reallocating resources toward underserved populations, provided that digital transformation does not exacerbate existing inequities through unequal access to technology [88,89].

### 7.7. Twin Transformation as a Strategic Framework for Specialty Evolution

Cardiothoracic surgery is at a pivotal moment in its evolution, shaped by rapid technological advancement and increasing environmental constraints. As a resource-intensive specialty, it must address the dual imperative of maintaining clinical excellence while reducing its ecological footprint. The twin transformation framework offers a strategic pathway for achieving this balance through the integrated adoption of digital health technologies and sustainability-driven practices [87].

Technologies such as artificial intelligence, digital twins, robotic systems, telemedicine, and interoperable electronic health records can enhance surgical precision, streamline workflows, and optimize resource use. Simultaneously, hospital-level sustainability initiatives, green procurement strategies, and environmentally conscious surgical practices reduce emissions and waste, aligning cardiothoracic care with ethical obligations and global health priorities [90]. While challenges related to cost, technical integration, and regulatory alignment persist, the evidence reviewed suggests that coordinated digital and sustainability efforts can generate meaningful clinical, operational, and environmental benefits. As the specialty continues to evolve, integrating innovation with sustainability will be essential to ensure that progress in patient care does not come at the expense of planetary and population health [91,92,93,94,95].

### 7.8. Translational and Clinical Implications: Realistic Integration and Areas of Over-Expectation

The twin transformation framework provides a conceptual lens for aligning digital innovation and sustainability in cardiothoracic surgery; however, its practical translation into routine clinical practice will likely occur incrementally rather than disruptively. Experience from digital health implementation studies suggests that integration into established clinical workflows, interoperability constraints, and clinician acceptance represent major determinants of real-world adoption [96,97]. Similarly, sustainability interventions in surgical settings tend to produce measurable impact when embedded within institutional governance structures rather than introduced as isolated initiatives [98].

In the near term, several developments appear realistically implementable. Artificial intelligence-assisted imaging analysis and perioperative risk stratification tools have demonstrated incremental predictive improvements in cardiac and thoracic populations, particularly when integrated into established electronic health record ecosystems [99,100]. Telemedicine-based follow-up and structured remote monitoring programs in cardiac surgery have been associated with reductions in early readmissions and improved post-discharge surveillance in selected cohorts [101,102]. From an environmental standpoint, operating room energy optimization, anesthetic gas substitution strategies, and rationalized surgical tray configurations have been shown to reduce carbon footprint and material waste without compromising safety [103,104]. These domains represent areas where digital and sustainability objectives can converge pragmatically.

In contrast, certain domains remain at risk of over-expectation. Although robotic-assisted surgery continues to expand, current systems remain surgeon-controlled telemanipulation platforms rather than autonomous decision-making technologies [105,106]. Likewise, digital twin models in cardiovascular medicine are still predominantly research tools, with limited prospective validation demonstrating outcome superiority over conventional planning approaches [107,108]. Furthermore, emerging literature cautions that digitalization does not automatically equate to sustainability gains, as data storage, high-performance computing, and device manufacturing carry their own environmental costs that are rarely incorporated into lifecycle assessments [109,110].

Accordingly, the translational trajectory of twin transformation should be guided by rigorous validation frameworks, transparent cost–benefit analyses, and standardized metrics capable of capturing clinical, economic, and environmental endpoints simultaneously. Calls for structured evaluation of artificial intelligence in healthcare emphasize prospective trials, external validation, and explainability as prerequisites for responsible clinical deployment [111,112]. A similarly cautious approach has been advocated in sustainable healthcare research, where harmonized carbon accounting methodologies and lifecycle analyses are necessary to avoid unintended environmental trade-offs [113]. Distinguishing between implementable optimization strategies and speculative technological promises is therefore essential to maintaining clinical credibility while fostering responsible innovation.

## 8. Limitations

Several limitations of this review should be acknowledged. First, the narrative nature of the review, rather than a systematic methodology, inherently constrains the comprehensiveness, reproducibility, and transparency of study selection. While a narrative approach was intentionally chosen to allow broader conceptual synthesis across emerging and heterogeneous domains, it does not follow predefined inclusion and exclusion criteria, structured quality appraisal, or formal risk-of-bias assessment typical of systematic reviews. Consequently, the conclusions presented should be interpreted as indicative and hypothesis-generating rather than definitive, and it is possible that relevant studies were not captured despite extensive database searching [114].

Second, the interdisciplinary scope of the reviewed literature—spanning cardiothoracic surgery, biomedical engineering, digital health, health informatics, and environmental sciences—introduces substantial heterogeneity in study designs, methodologies, outcome measures, and reporting standards. Differences in patient populations, clinical settings, institutional workflows, and levels of technological maturity further complicate direct comparison across studies. This heterogeneity limits the ability to synthesize findings quantitatively and necessitates cautious interpretation when extrapolating results or attempting to generalize conclusions across diverse healthcare environments.

Third, many components of the twin transformation framework, including digital twins, AI-assisted surgical planning, sustainable operating room practices, and extended reality-based training, remain at an early stage of clinical implementation. The majority of available evidence consists of pilot studies, retrospective analyses, proof-of-concept reports, or single-center experiences, with limited multicenter validation and scarce long-term outcome data. As a result, robust conclusions regarding clinical effectiveness, cost-efficiency, scalability, and environmental impact cannot yet be firmly established, underscoring the need for prospective, multicenter, and longitudinal research.

As a narrative review spanning clinical, technological, and environmental domains, this work synthesizes heterogeneous sources of evidence, including observational studies, pilot projects, modeling analyses, and policy documents. The rapidly evolving nature of digital health technologies and sustainability research, combined with variability in institutional practices, limits the ability to draw definitive quantitative conclusions. Accordingly, the aim of this review is conceptual integration rather than meta-analytic synthesis.

Finally, the applicability and feasibility of twin transformation strategies are highly context dependent. High-resource healthcare systems with advanced digital infrastructure, dedicated sustainability programs, and supportive policy environments may experience different benefits and challenges compared with low- and middle-income settings, where financial constraints, limited digital connectivity, and workforce shortages may restrict implementation. Regulatory frameworks, reimbursement models, and institutional priorities also vary widely across regions, influencing the adoption and sustainability of these approaches. Therefore, successful implementation of twin transformation principles will require local adaptation, context-specific evaluation, and flexible implementation strategies rather than a one-size-fits-all model [115].

## 9. Conclusions

Cardiothoracic surgery faces the dual imperative of sustaining rapid technological progress while reducing its considerable environmental impact. This review highlights twin transformation—the integrated application of digital innovation and sustainability—as a pragmatic and forward-looking framework to address this challenge. Evidence suggests that digital tools such as artificial intelligence, digital twins, robotics, telemedicine, and interoperable health records can enhance precision, efficiency, and personalization of care, while simultaneously supporting resource optimization and emission reduction when aligned with sustainable surgical practices and institutional policies. Although current evidence is largely preliminary, the convergence of digitalization and sustainability offers meaningful clinical, operational, and ethical benefits. Advancing this paradigm will require standardized outcome metrics, robust multicenter validation, supportive governance, and context-specific implementation strategies. Embracing twin transformation positions cardiothoracic surgery to lead the transition toward high-quality, technologically advanced, and environmentally responsible surgical care. Importantly, the twin transformation framework remains primarily conceptual at present. Future research should move beyond feasibility and pilot studies toward standardized outcome metrics capable of simultaneously capturing clinical, economic, and environmental endpoints across diverse healthcare systems.

## Figures and Tables

**Table 1:** Key domains of digital transformation in cardiothoracic surgery across the perioperative continuum.

Digital Technology	Main Applications	Stage of Care	Key Benefits	References
Artificial intelligence & big data	Imaging analysis; risk prediction; workflow optimization	Pre-, intra-, post-operative	Improved accuracy; personalized risk; efficiency gains	[15,16,17,18,19,20,21,22]
Robotic surgical systems	RATS; robotic CABG; valve repair	Intraoperative	Less invasiveness; faster recovery; higher precision	[23,24,25,26,27]
Minimally invasive techniques	VATS; hybrid coronary procedures	Intraoperative	Reduced morbidity; shorter LOS	[24,25,26,27]
Digital twins	Patient-specific modeling; surgical planning	Preoperative	Personalized planning; reduced uncertainty	[29,30,31,65]
VR/AR/MR (XR)	Simulation training; intraoperative visualization	Training; perioperative	Safer training; reduced physical resources	[28,61,62,63]
Telemedicine & RPM	Virtual follow-up; complication monitoring	Postoperative	Fewer readmissions; reduced travel	[31,32,33,34]
EHRs & interoperability	Data integration; predictive analytics	All stages	Better coordination; reduced redundancy	[35,36,37,38]
Digital perioperative platforms	Scheduling; logistics; analytics	Perioperative	Optimized workflows; resource efficiency	[60,73]

Abbreviations: AR, augmented reality; CABG, coronary artery bypass grafting; EHR, electronic health record; LOS, length of stay; MR, mixed reality; RATS, robotic-assisted thoracic surgery; RPM, remote patient monitoring; VATS, video-assisted thoracic surgery; VR, virtual reality; XR, extended reality (encompassing VR, AR, and MR).

**Table 2:** Major environmental challenges associated with cardiothoracic surgery and corresponding mitigation strategies at the operating room and institutional levels.

Sustainability Challenge	Primary Source of Impact	Mitigation Strategy	Expected Benefit	References
High operating room energy use	HVAC systems; lighting; long procedures	Energy-efficient OR design; optimized HVAC	Reduced energy consumption; lower CO_2_ emissions	[45,46,47,48,54]
Anesthetic gas emissions	Desflurane; nitrous oxide	Low-impact anesthetic protocols	Reduced greenhouse gas emissions	[45,50]
Excess disposable waste	Single-use instruments; drapes; tubing	Reusable instruments; waste segregation	Reduced landfill waste; lower lifecycle emissions	[51,52,53]
Inefficient instrument use	Overprepared trays; unused supplies	Lean workflows; tray optimization	Reduced material use; cost savings	[51,52]
Supply chain inefficiencies	Overstocking; logistics redundancy	Sustainable procurement policies	Lower resource use; reduced emissions	[49,54]
Hospital-level emissions	Infrastructure; staff commuting	Renewable energy; green hospital initiatives	System-wide decarbonization	[48,54]
Inequitable access to care	Patient travel; geographic barriers	Telemedicine integration	Lower travel emissions; improved access	[31,32,33,34,59]

Abbreviations: CO_2_, carbon dioxide; HVAC, heating, ventilation, and air conditioning; OR, operating room.

**Table 3:** Examples of synergistic interactions between digital health technologies and sustainability objectives in cardiothoracic surgery, illustrating the twin transformation framework.

Digital Intervention	Sustainability Driver	Mechanism of Synergy	Clinical Impact	Environmental Impact	References
AI-driven OR scheduling	Energy efficiency	Reduced idle time and over-preparation	Improved workflow efficiency	Lower energy consumption	[60,73]
Telemedicine & RPM	Emission reduction	Reduced patient travel	Improved follow-up; fewer readmissions	Lower transport-related emissions	[31,32,33,34,64]
Digital twins	Resource optimization	In silico planning and prediction	Fewer complications; personalized care	Reduced waste and reinterventions	[29,30,31,65]
VR/XR-based training	Material reduction	Virtual simulation replaces physical models	Safer skills acquisition	Reduced training-related waste	[61,62,63]
EHR-integrated analytics	Overuse reduction	Avoidance of redundant tests	Better decision-making	Lower resource utilization	[35,36,37,38]
Robotic/minimally invasive surgery	Shorter hospitalization	Reduced tissue trauma	Faster recovery	Lower resource and energy use	[23,24,25,26,27,49]

Abbreviations: AI, artificial intelligence; EHR, electronic health record; OR, operating room; RPM, remote patient monitoring; VR, virtual reality; XR, extended reality.

## Data Availability

No new data were created or analyzed in this study. Data sharing is not applicable to this article.
